# Solid State Structure and Solution Thermodynamics of Three-Centered Hydrogen Bonds (O∙∙∙H∙∙∙O) Using *N*-(2-Benzoyl-phenyl) Oxalyl Derivatives as Model Compounds

**DOI:** 10.3390/molecules190914446

**Published:** 2014-09-12

**Authors:** Carlos Z. Gómez-Castro, Itzia I. Padilla-Martínez, Efrén V. García-Báez, José L. Castrejón-Flores, Ana L. Peraza-Campos, Francisco J. Martínez-Martínez

**Affiliations:** 1 Departamento de Ciencias Básicas, Unidad Profesional Interdisciplinaria de Biotecnología del Instituto Politécnico Nacional, Av. Acueducto s/n Barrio la Laguna Ticomán, México D.F. 07340, Mexico; E-Mails: carloszepgc@gmail.com (C.Z.G.-C.); efren1003@yahoo.com.mx (E.V.G.-B.); j_luis666@msn.com (J.L.C.-F.); 2 Laboratorio de Posgrado, Facultad de Ciencias Químicas, Universidad de Colima, Km 9 Carretera Colima-Coquimatlán, Colima 28400, Mexico; E-Mail: peraza@ucol.mx

**Keywords:** three-center hydrogen bond, oxalamide, oxalamate, steric effect, solvent effect, proton mobility, cooperativity

## Abstract

Intramolecular hydrogen bond (HB) formation was analyzed in the model compounds *N*-(2-benzoylphenyl)acetamide, *N*-(2-benzoylphenyl)oxalamate and *N^1^*,*N^2^*-bis(2-benzoylphenyl)oxalamide. The formation of three-center hydrogen bonds in oxalyl derivatives was demonstrated in the solid state by the X-ray diffraction analysis of the geometric parameters associated with the molecular structures. The solvent effect on the chemical shift of H6 [δH6(DMSO-*d*_6_)–δH6(CDCl_3_)] and Δδ(ΝΗ)*/*Δ*T* measurements, in DMSO-*d*_6_ as solvent, have been used to establish the energetics associated with intramolecular hydrogen bonding. Two center intramolecular HB is not allowed in *N*-(2-benzoylphenyl)acetamide either in the solid state or in DMSO-*d*_6_ solution because of the unfavorable steric effects of the *o*-benzoyl group. The estimated Δ*Hº* and Δ*Sº* values for the hydrogen bonding disruption by DMSO-*d*_6_ of 28.3(0.1) kJ·mol^−1^ and 69.1(0.4) J·mol^−1^·K^−1^ for oxalamide, are in agreement with intramolecular three-center hydrogen bonding in solution. In the solid, the benzoyl group contributes to develop 1-D and 2-D crystal networks, through C–H∙∙∙A (A = O, π) and dipolar C=O∙∙∙A (A = CO, π) interactions, in oxalyl derivatives. To the best of our knowledge, this is the first example where three-center hydrogen bond is claimed to overcome steric constraints.

## 1. Introduction

Non-covalent interactions have been considered as the driving forces involved in molecular recognition processes. Among them, hydrogen bonding is probably the most important in biological systems [1,2]. Oxalamate and oxalamide moieties, in contrast to amides, are not found in biomolecules, but they have been employed as pseudopeptide templates in bioorganic and medicinal chemistry [3,4]. Bis-oxalamides exhibit gelation properties, and such ability is a consequence of strong and directional intermolecular hydrogen bonding [5].

**Figure 1:**
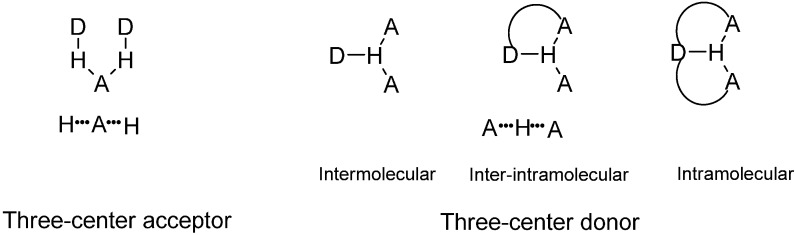
Three-center hydrogen bond.

The three-centered hydrogen bond (THB), or bifurcated hydrogen bond, classified as non-classical hydrogen bonding, is responsible for the biological activity of peptides, proteins, DNA, and other bioactive molecules [6,7,8]. One acceptor and two donor groups (H∙∙∙A∙∙∙H) or one donor and two acceptor groups (A∙∙∙H∙∙∙A) can form two structurally different THBs (Figure 1) [9,10], known as three-center acceptor (bifurcated acceptor) and three-center donor (bifurcated donor) hydrogen bonds, respectively. In addition, they can be composed of intermolecular, intramolecular or both components. The energetic superiority of two-centered or regular HB over a bifurcated acceptor THB in non- aromatic systems has been demonstrated [11,12], but over a bifurcated donor THB is still in a matter of debate [13,14,15].

From a supramolecular point of view, intermolecular H∙∙∙A∙∙∙H THBs have been exploited to form multicomponent crystals [16,17,18], they have been associated with the properties of amyloid fibrils [19] and their reversible formation has been induced by high pressure phase transition in organic crystals [20]. On the other hand, intramolecular A∙∙∙H∙∙∙A THBs have been successfully used to rigidify the backbones of macrocyclic precursors [21] as well as for promoting the formation of stable dimeric complexes in solution [22]. A complete description of the effect of THB formation on the properties of several materials has been compiled elsewhere [23].

**Figure 2:**
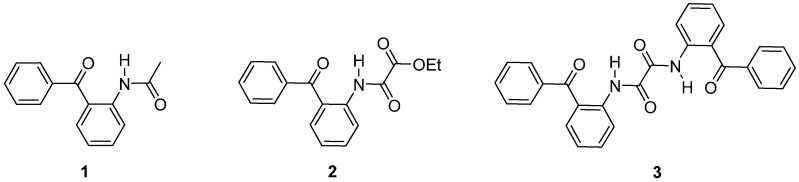
Model compounds.

In this contribution, *N*-(2-benzoylphenyl)acetamide (**1**) has been chosen as a model compound for a two centered or regular hydrogen bond (HB), ethyl *N*-(2-benzoylphenyl)oxalamate (**2**) for a three-centered hydrogen bond (THB) and *N^1^*,*N^2^*-bis(2-benzoylphenyl)oxalamide (**3**) as a model compound for cooperativity effects associated with THB formation (Figure 2). It is worth mentioning that in a previous publication [24], we have used an analogous approach to study the thermodynamic properties and cooperativity involvement in THB formation in solution. Herein, the model compounds present important steric effects exerted by the benzoyl group not included before in the study of THBs.

Compound **1** has been used as a model compound of ATPasa binding [25], and as starting material for the synthesis of more complex compounds such as quinazolines [26,27], indole derivatives [28,29,30], 4-phenylquinolin-2(1*H*)-one derivatives [31], azabenzopyrylium perchlorates [32] and COX inhibitors [33]. Compounds **2** and **3** have been used as starting materials for the synthesis of indole derivatives [34,35], while compound **3** has been additionally used to form complexing ligands for several metals [36,37,38]. Surprisingly, despite the wide use of these compounds their X-ray structures have not been reported yet. Therefore, the aims of this contribution were to establish the molecular structure of the model compounds in the solid state by X-ray diffraction analysis and to estimate the thermodynamic properties associated with hydrogen bonding in solution by NMR.

## 2. Results and Discussion

### 2.1. Molecular Structure of **1**–**3** in the Solid State

**Figure 3:**
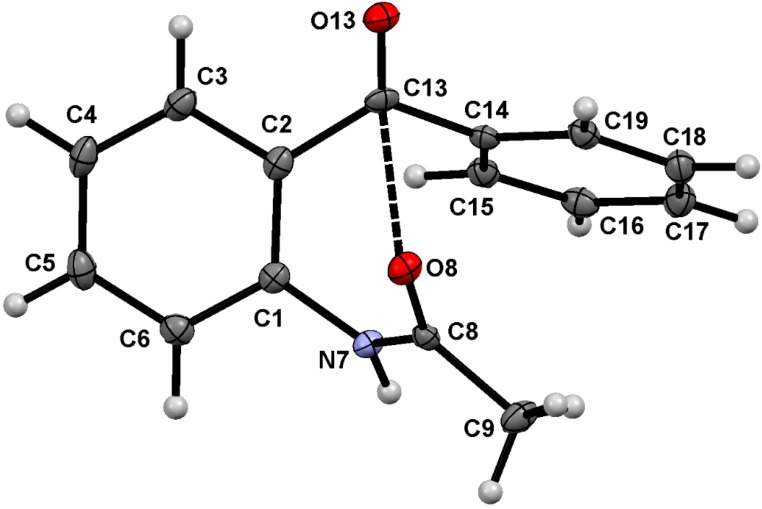
Molecular structure of compound **1**. ORTEP at 30% probability level.

**Figure 4:**
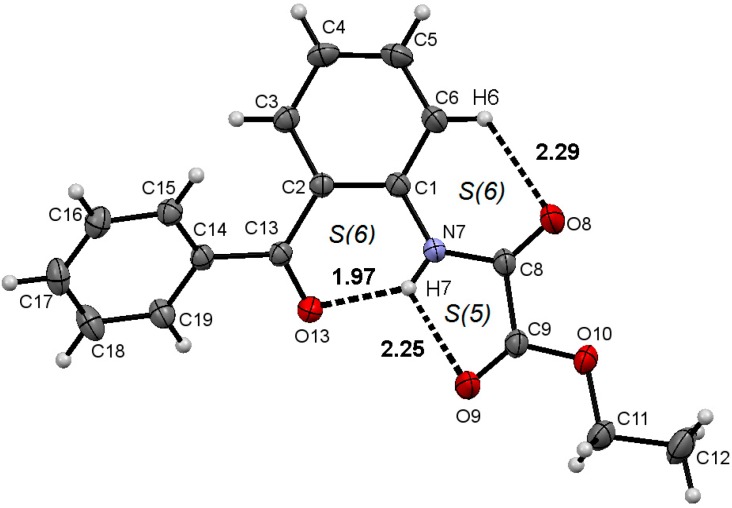
Molecular structure of compound **2**. ORTEP at 30% probability level.

**Figure 5:**
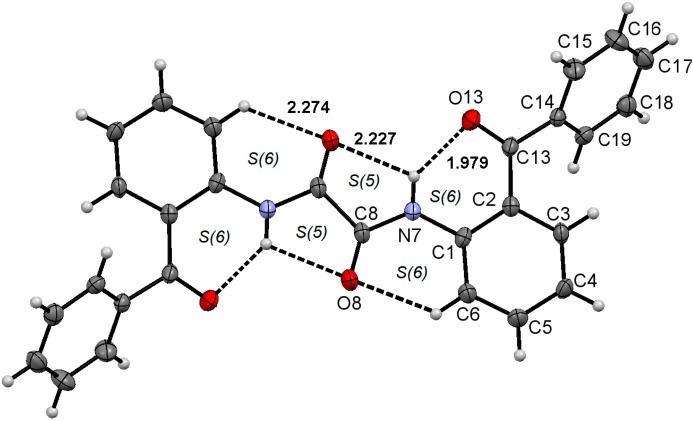
Molecular structure of compound **3**. ORTEP at 30% probability level.

The molecular structures and atom numbering of acetamide **1**, oxalamate **2** and oxalamide **3** are shown in Figure 3, Figure 4 and Figure 5, respectively. A summary of bond lengths and angles is listed in Table 1. The bond lengths N7–C1 and N7–C8, around the benzamide group, are very similar between the three compounds, and the mean values are very close to the ones measured for acetanilide [1.413(3) Å and 1.354(3), respectively] [39,40,41]. The amide group in compound **1** is out of the plane of the phenyl ring, whereas the ethyloxalamate group in compound **2** and oxalamide group in compound **3** are almost in the same plane as the phenyl ring, as shown by the C2–C1–N7–C8 torsion angles. It is worthy of mention that the structurally analogous compounds *N*-(2-acetylphenyl)acetamide [42] and *N^1^*,*N^2^*-bis(2-acetylphenyl)oxalamide [43,44] adopt a planar conformation forming two- and three-centered hydrogen bonds in the solid state.

The two carbonyl groups of the oxalyl moiety in compounds **2** and **3** are antiperiplanar, with O8=C8–C9(8a)=O9(8a) torsion angles near to 180°, in accord with the most frequently adopted conformation seen in open systems. In spite of the planarity exhibited by the oxalamate and oxalamide groups, the CO–CO bond length values for **2** and **3** are close to the value for C*sp^3^*–C*sp^3^* single bond [45], indicating the absence of π conjugation.

**Table 1:** Selected bond lengths and angles of compounds **1**–**3**.

	1	2	3
Atoms	Bond Lengths (Å)		
O8–C8	1.234(2)	1.204(3)	1.211(4)
O13–C13	1.217(2)	1.227(2)	1.223(5)
N7–C1	1.425(2)	1.399(2)	1.402(4)
N7–C8	1.338(2)	1.346(2)	1.350(4)
C1–C2	1.398(2)	1.416(3)	1.407(4)
C2–C13	1.491(2)	1.486(2)	1.483(5)
C8–C9(8a)	1.502(2)	1.538(3)	1.548(5)
C13–C14	1.490(3)	1.496(2)	1.495(5)
	**Bond and Torsion Angles (°)**		
C1–N7–C8	123.27(14)	129.61(16)	128.9(3)
N7–C1–C2	121.49(14)	119.08(16)	118.8(3)
C2–C1–N7–C8	52.3(2)	−171.96(18)	−174.7(3)
O8–C8–N7–C1	−3.2(3)	−5.8(3)	−1.2(6)
O8–C8–C8a–O8a		166.7(2)	−180.0(3)
O13–C13–C14–C15	−135.18(17)	151.65(18)	−40.2(4)
C1–C2–C13–O13	−145.02(17)	25.4(3)	19.14(4)

Most benzamides bearing hydrogen bonding acceptor groups in the *ortho* position form intramolecular hydrogen bonds with the amide NH [46]. In this case, however, the carbonyl moiety of the 2-benzoyl pendant group is almost opposite the NH, [C1–C2–C13–O13 = −145.21(16)°] avoiding intramolecular hydrogen bonding in amide **1**, but instead, allowing intermolecular hydrogen bonding (*vide infra*) and intermolecular dipolar carbonyl-carbonyl interactions (C8O8∙∙∙C13 distance of 2.826(2) Å, C8–O8∙∙∙C13 angle of 91.8(2)°). This conformational preference can be explained by the steric effect of the 2-benzoyl group that restricts the free rotation around the C2–CO bond. In contrast to the result obtained for amide **1**, the 2-benzoyl pendant group points towards the NH in both oxalamate **2** and oxalamide **3**, with small C1–C2–C13–O13 torsion angles, forming an intramolecular hydrogen bond. The gradual narrowing of the N7–C1–C2 angle is the results of the conformation exhibited by each compound. In fact, the NH becomes engaged with two carbonyl acceptors, forming an intramolecular three centered hydrogen bonding motif *S*(6) type [47], whose geometric parameters are listed in Table 2. In addition, the secondary soft H-bonding interaction C6–H6∙∙∙O8 completes the H-bonding pattern as an *S*(6)*S*(5)*S*(6) and [*S*(6)*S*(5)*S*(6)]_2_ motifs for **2** and **3**, respectively. In oxalamate and bis-oxalamide systems, the participation of C6–H6∙∙∙O8 hydrogen bonding as a cooperative interaction in THB formation, has not been noticed in either solution or the solid state [48,49].

The whole H-bonding system forms a plane defined by the O13C13C2C1C6N7C8O8C9(8a)O9(8a) group of atoms of which the maximum deviation is exhibited by O13 and O9 [−0.3240 (13) and 0.2808 (17) Å, respectively], in compound **2**, and C13 and O13 [−0.1804 (33) and 0.1356 (26) Å, respectively], in compound **3**. These results suggest that the formation of the intramolecular three-centered hydrogen bond O9(8a)∙∙∙H7∙∙∙O13, in compounds **2** and **3**, is strongly favored over an intermolecular one, as observed in compound **1**.

**Table 2:** Hydrogen bonding geometry.

Comp.	D–H∙∙∙A	Symmetry Code	D–H (Å)	H∙∙∙A (Å)	D∙∙∙A (Å)	D–H∙∙∙A (°)
**1**	N7–H7∙∙∙O8	−½+x, 1−y, −½+z	0.88	1.91	2.7491(19)	160
	C17–H17∙∙∙O8	−1+x, y, z	0.95	2.50	3.439(2)	170
	C4–H4∙∙∙ *Cg*(2)	½+x, −y, −½+z		2.54	3.4104(19)	153
**2**	N7–H7∙∙∙O9		0.86	2.25	2.666(2)	110
	N7–H7∙∙∙O13		0.86	1.97	2.662(2)	136
	C6–H6∙∙∙O8		0.93	2.29	2.908(3)	124
	C17–H17∙∙∙O8	−1+x, y, −1+z	0.93	2.57	3.391(3)	147
**3**	N7–H7∙∙∙O8a	−x, −y, 1−z	0.86	2.23	2.665(4)	111
	N7–H7∙∙∙O13		0.86	1.98	2.673(4)	137
	C6–H6∙∙∙O8		0.93	2.27	2.900(4)	124
	C4–H4∙∙∙*Cg*(2)	1−x, −y, −z		2.81	3.612(4)	145

The sum of angles around H7 = 360.0° and 359.0° for compounds **2** and **3**, respectively.

### 2.2. Supramolecular Structure of **1**–**3** in Solid State

**Figure 6:**
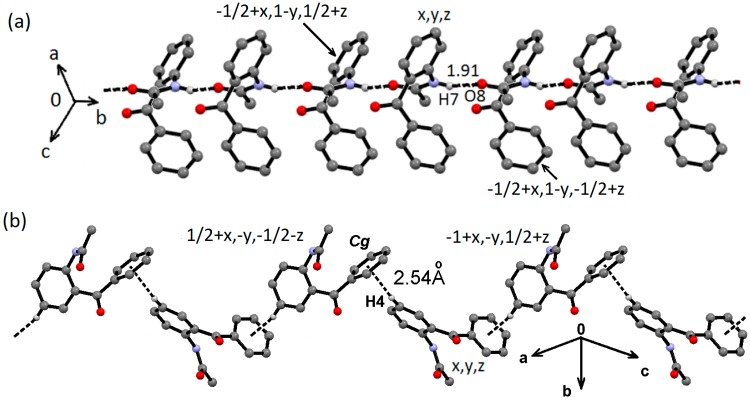
Supramolecuar structure of compound **1**, (**a**) through N7–H7···O8 and (**b**) C4–H4∙∙∙*Cg*(2) interactions.

The supramolecular structure of amide **1** is achieved by a combination of strong and weak hydrogen bonding interactions, whose geometric parameters are listed in Table 2. The amide NH is involved in strong H-bonding interactions with the amide carbonyl from a neighboring molecule, N7–H7···O8, developing a *C*(4) chain along the [1 0 −1] direction (Figure 6a). The second dimension is accomplished by C4–H4∙∙∙*Cg*(2) weak interactions (*Cg*(2) is the centroid of the C14–C19 ring) along the [1 0 1] direction (Figure 6b).

**Figure 7:**
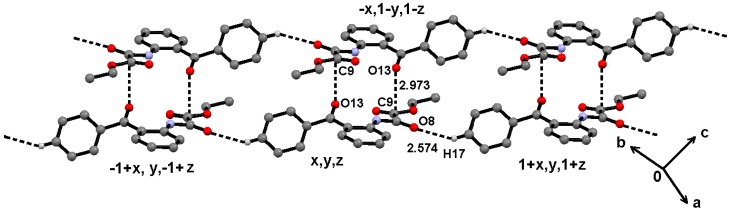
Supramolecuar structure of compound **2**, through C13O13∙∙∙C9 and C17–H17∙∙∙O8 interactions.

Because the N7–H7, the best H-donor, is involved in intramolecular hydrogen bonding, the supramolecular structure is governed by soft C–H∙∙∙A (A = O, π) [50] and dipolar C=O∙∙∙A (A = CO, π) interactions [51] for **2** and **3**, and the hydrogen bonding geometric parameters are listed in Table 2. In the case of oxalamate **2**, the C13–O13∙∙∙C9 dipolar interaction between ketone and ester carbonyls (C13O13∙∙∙C9 distance of 2.973(3) Å; C13–O13∙∙∙C9 angle of 112.5(2)°; symmetry code: −x, 1−y, 1−z), forms centrosymmetric dimers interlinked by C17–H17∙∙∙O8 interactions to form ribbons that propagate along the [1 7 −1] direction (Figure 7).

**Figure 8:**
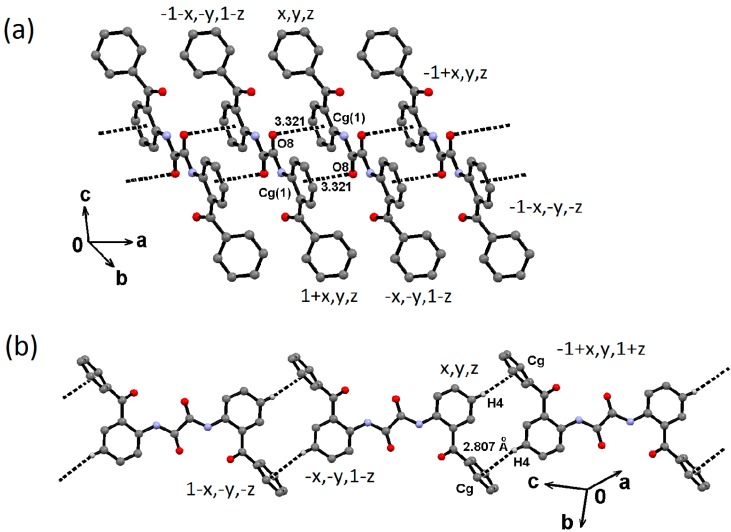
Supramolecuar structure of compound **3**, π-stacking and tape formation through (**a**) C8O8∙∙∙*Cg*(1) and (**b**) C4–H4∙∙∙ * Cg*(2) interactions, respectively.

Molecules of oxalamide **3** π-stack along the [0 2 3] direction through C8–O8∙∙∙*Cg*(1) interactions [O8∙∙∙*Cg*(1) distance of 3.321(3) Å; C8–O8∙∙∙*Cg*(1) angle of 86.12(19); symmetry code: 1−x, −y, 1−z; *Cg*(1) is the centroid of the benzamide (C1–C6) ring] (Figure 8a). The stacked columns are joined together through C4–H4∙∙∙*Cg*(2) interactions along the [2 7 2] direction (Figure 8b).

### 2.3. Molecular Structure of **1**–**3** in Solution

Compounds **1**–**3**, were characterized by ^1^H- and ^13^C-NMR; nOe experiments on the NH proton were used to unequivocally assign H6. A summary of data concerning HB is given in Table 3. The NHCO carbon atom of amide **1** appears at higher frequency when compared with compounds **2** and **3**. These results suggest the gradual increase of electronic delocalization of the nitrogen lone pair into the adjacent carbonyl group in going from **1** to **3**, in agreement with the smaller N7–C8 distance than the N7–C1 one observed by X-ray diffraction (*vide supra*). The conformation observed in the solid state seems to be the same in DMSO-*d*_6_ solution for each compound, and the chemical shift of H6 is very sensitive to the field effects caused by the neighboring amide carbonyl group. In DMSO-*d*_6_ solution, the δH6 is shifted to high frequencies by the local deshielding effect of the amide carbonyl group. This effect has been known for a long time for *o*-substituted anilides [52]. Thus, the amide CO is found most of the time in the nearby of H6, in *exo* conformation, in oxalamide **3** followed by oxalamate **2** and amide **1**. On the other hand, the chemical shift of the NH also appears to higher frequency, indicating a progressive engagement in HB in the compounds in the order **3** > **2** > **1**.

**Table 3:** Summary of ^1^H- and ^13^C-NMR data for compounds **1**–**3** relevant to hydrogen bonding.

Comp.	(DMSO-*d*_6_)				(CDCl_3_)		ΔδNH ^b^	ΔδH6 ^c^
	δCO	δNH	δH6	−ΔδNH/ΔT ^a^	δNH	δH6		
**1**	169.2	10.1	7.65	2.59	10.8	8.63	−0.7	−0.98
**2**	155.6	11.5	8.06	2.67	12.3	8.71	−0.8	−0.65
**3**	158.4	11.7	8.23	1.88	12.5	8.81	−0.8	−0.58

^a^ ppb·K^−1^; ^b^ [δNH(DMSO)–δNH(CDCl_3_)]; ^c^ [δH6(DMSO)–δH6(CDCl_3_)].

The solvent effect on the NH chemical shift has been accepted as a general test for proton mobility [53]. Thus, the ^1^H-NMR spectra of compounds **1**–**3** were recorded in CDCl_3_ solution and compared with those recorded in DMSO-*d*_6_ solution, and the results are shown in Table 3 for NH and H6. In general, both δNH and δH6 signals, in CDCl_3_ solutions, are shifted to higher frequencies relative to DMSO solutions. Small and similar ΔδNH values [δNH(DMSO)–δNH(CDCl_3_)] were observed for compounds **1**–**3**. This result suggests that the δNH is not sensitive to solvent effects, probably due to the steric protection effect exerted by the benzoyl group. Additionally, the δH6, in CDCl_3_ solutions, is shifted to high frequencies in the three compounds with values of ΔδH6 decreasing in the following order: **1** > **2** > **3**. These results indicate that the conformation *exo*, of the amide carbonyl, is favored in less polar solvents and the effect is smaller for strongly hydrogen bonded systems. Moreover, in chloroform solutions the three model compounds are in a full hydrogen bonding state.

A more reliable criterion to judge the strength of HB is the temperature gradient of the amide proton chemical shift. Smaller ΔδNH/ΔT values are associated with strong intramolecular HB particularly those values smaller than −2.0 ppb·K^−1^ that correspond with three centered HB systems [54]. Thus, the measured values for compounds **1**–**3**, shown in Table 3, are in agreement with the formation of a regular hydrogen bond in **1** and **2** and with the presence of a three centered hydrogen bond in compound **3** in DMSO-*d*_6_ solution.

Furthermore, the Δδ(ΝΗ)*/*Δ*T* measurements, using DMSO-*d*_6_ as solvent, have been used to establish the energetics associated with intramolecular hydrogen bonding [24,55]. The method is based on a nonlinear fitting (NLF) procedure, followed by van't Hoff data treatment which is applied to the equilibrium established between an intramolecular hydrogen bonding at the initial state and the association with the solvent at the final state of the NH proton, at increasing temperatures. The results are listed in Table 4 as well as the corresponding values for acetanilide, ethyl *N*-phenyloxalamate and *N^1^*,*N^2^*-bis(2-nitrophenyl)oxalamide, as reference compounds [24]. In each case both enthalpy and entropy changes are positive, in agreement with the energy required to disrupt intramolecular hydrogen bonding and to reach a more disordered final state by association with the solvent. Both Δ*Hº* and Δ*Sº* values for amide **1** are in the range for non-hydrogen bonding state, but they are smaller than those predicted for acetanilide. In the case of compound **2**, the similarity of its Δ*Hº* and Δ*Sº* values with those previously reported for ethyl *N*-phenyloxalamate suggests the same HB scheme characterized by the absence of of intramolecular HB with the *o*-carbonyl moiety. The predicted Δ*Hº* and Δ*Sº* values, for compound **3**, suggest a full three centered hydrogen bonding scheme which is stronger than the one found in *N^1^*,*N^2^*-bis(2-nitrophenyl)oxalamide.

For reference purposes, the estimated Δ*H°* and Δ*S°* values for oxalamates, 18.7(1.0) kJ·mol^−1^ and 42.0(4.7) J·mol^−1^·K^−1^ and oxalamides, 24.4(1.7) kJ·mol^−1^ and 61.9(11) J·mol^−1^·K^−1^, are given. These values represent the energy required to break a full three-centered hydrogen bond (A∙∙∙H∙∙∙O=C) [24].

**Table 4:** Thermodynamic parameters Δ*Hº* and Δ*Sº* (at 298.15 K) of the amide N–H∙∙∙DMSO-*d*_6_ HB formation and their standard errors obtained from van’t Hoff plots for compounds **1**–**3** and reference values for acetanilide, ethyl *N*-phenyl oxalamate and *N^1^*,*N^2^*-bis(2-nitrophenyl)oxalamide.

Comp.	Δ *Hº*/kJ·mol^−1^	Δ *Sº*/J·mol^−1^·K^−1^
Acetanilide [24]	9.57(0.02)	16.68(0.07)
Ethyl *N*-phenyl oxalamate [24]	11.82(0.08)	23.2(0.2)
*N^1^*,*N^2^*-bis(2-nitrophenyl)oxalamide [24]	21.1(0.2)	58.1(0.5)
**1**	6.60(0.03)	12.9(0.1)
**2**	12.10(0.08)	23.7(0.2)
**3**	28.3(0.1)	69.1(0.4)

## 3. Experimental Section 

### 3.1. Instruments

Melting points were measured on an Electrothermal IA apparatus and are uncorrected. IR spectra were recorded at 25 °C using a Varian 3100 FT-IR with ATR system Excalibur Series spectrophotometer. ^1^H- and ^13^C-NMR spectra was recorded on a Varian Mercury 300 MHz (^1^H, 300.08; ^13^C, 75.46 MHz) in DMSO-*d*_6_. The spectra was measured with tetramethylsilane as internal reference following standard techniques. Standard HETCOR and nOe experiments were performed to properly assign H6. Variable temperature experiments were performed using a temperature controller that maintained the temperature constant within 0.2 °C and increased the temperature automatically by 10 °C increments, from 20 to 120 °C, with a delay of 5 min for the temperature stabilization. Each spectrum was obtained with 32 scans. Samples concentration was maintained at 5 mg/0.4 mL or less in DMSO-*d*_6_ solutions. Temperature controller was calibrated using standard techniques given by the manufacturer. General procedure for NLF and van’t Hoff data treatments were performed as reported [21,51].

General Crystallographic data (excluding structure factors) for the structures in this paper have been deposited in the Cambridge Crystallographic Data Centre as supplementary publication numbers CCDC **1** (1013358), **2** (1013356) and **3** (1013357). A summary of collection and refinement of the X-ray data is listed in Table 5. H atoms were treated as riding atoms, with C–H distances in the range of 0.93–0.96 Å and N–H distances in the range of 0.86–0.88 Å. X-ray diffraction cell refinement and data collection: BRUKER APEX II Diffractometer with Mo radiation (kα, λ = 0.71073 Å) and SAINT [56], programs used to solve structures: SHELXS-97 [57], software used to prepare material for publication: PLATON [58], *WinGX* [59] and Mercury [60].

**Table 5:** Collection and refinement X-ray data of compounds **1**–**3**.

Compounds	1	2	3
CCDC number	1013358	1013356	1013357
Formula	C_15_H_13_N_1_O_2_	C_17_H_15_N_1_O_4_	C_28_H_20_N_8_O_4_
M (g·mol^−1^)	252.0	297.3	448.5
Crystal system	Monoclinic	Monoclinic	Monoclinic
Space group	P n	P 2_1_/c	P 2_1_/c
a (Å)	8.3679(9)	9.3796(8)	6.2377(6)
b (Å)	9.1351(10)	15.2202(13)	17.7892(18)
c (Å)	8.9361(9)	10.6317(9)	10.8548(9)
α (°)	90	90	90
β (°)	115.320(2)	102.815(1)	114.429(4)
γ (°)	90	90	90
V (Å^3^)	618.94(7)	1478.41(4)	1096.6(2)
Z	2	4	2
ρ_calcd._ (g·cm^−3^)	1.324	1.34	1.36
μ (mm^−1^)	0.086	0.096	0.092
F (000)	252.0	623.9	467.9
Crystal size (mm)	0.50 × 0.50 × 0.40	0.36 × 0.30 × 0.28	0.40 × 0.30 × 0.30
Temp. (K)	100 (2)	293(2)	100(2)
θ range (°)	2.2–26.0	2.2–27.6	2.3–25.0
Reflections collected	6206	16595	9547
Independent reflections	2419	3381	1930
Data/restraints/parameters	2419/2/163	3381/0/199	1930/0/154
Goof	1.139	1.216	1.148
R (int)	0.030	0.029	0.068
Final R indices [I > 2σ(I)], R^1^/wR^2^	0.035/0.090	0.066/0.150	0.077/0.142
Largest diff. peak/hole (e·Å^−3^)	0.284/−0.222	0.230/−0.258	0.201/−0.185

### 3.2. Synthesis of Compounds

Compounds **1**–**3** are known. They were synthetized starting from commercial 2-aminobenzophenone and the corresponding acid chlorides (acetyl chloride (**1**), monoethyl oxalyl chloride (**2**) or oxalyl chloride (**3**)) in equimolar quantities following reported procedures [48]. All of the compounds were crystallized from CHCl_3_ solutions.

## 4. Conclusions 

The formation of two- and three-center hydrogen bonds in the solid state was demonstrated by the analysis of the geometric parameters associated with the molecular structure by X-ray diffraction. The combined effects of N–H∙∙∙O=CPh, C6–H∙∙∙O=C and N–H∙∙∙O=C interactions form THBs and have a global effect resulting in the flattening of the molecular structure. Thus, the two center intramolecular HB is not allowed in *N*-(2-benzoylphenyl)acetamide neither in the solid state nor in DMSO-*d*_6_ solution, and this effect is attributed to the unfavorable steric effects of *o*-benzoyl group.

The *syn*-disposition between the NH and ester carbonyl in ethyl *N*-(2-benzoylphenyl)oxalamate promotes the alignment of the NH, facilitating the hydrogen bonding engagement with the carbonyl moiety of the benzoyl group in the solid state but not in DMSO solution. Cooperative stabilizing effects are necessary to overcome the steric constraints imposed by the benzoyl group allowing the formation of THB in *N^1^*,*N^2^*-bis(2-benzoylphenyl)oxalamide in either solid state and in DMSO solution. Extensive intramolecular hydrogen bonding, when at least six adjacent rings [*S*(6)*S*(5)*S*(6)]_2_ are formed, are required to yield the steric constraints imposed by the benzoyl group in solution, whereas in the solid the formation of intermolecular hydrogen bonding system *S*(6)*S*(5)*S*(6) is sufficient.

Due to the steric restrictions, the effect of the solvent as a test for proton mobility on the NH chemical shift analysis is not accurate. However, the compounds showed intramolecularly hydrogen bonding in chloroform solutions in agreement with the solvent effect observed on the aromatic proton H6 of ca 1.0 ppm.

The estimated Δ*Hº* and Δ*Sº* values for the hydrogen bonding disruption in DMSO-*d*_6_ are 28.3(0.1) kJ·mol^−1^ and 69.1(0.4) J·mol^−1^·K^−1^ for oxalamide **3**, which correlates with the energy of the intramolecular three-center hydrogen bonding in solution demonstrating that it is thermodynamically favored.

The benzoyl group contributes to develop 1-D and 2-D crystal networks, in compounds **2** and **3**, built by the concurrence C–H∙∙∙A (A = O, π) and dipolar C=O∙∙∙A (A = CO, π) interactions. In contrast, most *ortho*-substituted oxalamides are characterized by their lack of supramolecular structure.
